# Randomized crossover comparison of two teriparatide self-injection regimens for primary osteoporosis: final report of the Japanese Osteoporosis Intervention Trial 06 (JOINT-06)

**DOI:** 10.1007/s00774-025-01631-w

**Published:** 2025-09-03

**Authors:** Sakae Tanaka, Yukari Uemura, Shiro Tanaka, Yasuhiro Takeuchi, Naoto Endo, Junichi Takada, Satoshi Ikeda, Jun Iwamoto, Nobukazu Okimoto, Satoshi Soen

**Affiliations:** 1 Department of Orthopaedic Surgery, Faculty of Medicine, The University of Tokyo, 7-3-1 Hongo, Bunkyo-ku, Tokyo, 113-8655 Japan; 2 Biostatistics Section, Department of Data Science, Center for Clinical Sciences, Japan Institute for Health Security, Toyama, Shinjuku-ku, Tokyo, Japan; 3 Department of Clinical Biostatistics, Graduate School of Medicine, Kyoto University, Yoshida Konoe-cho, Sakyo-ku, Kyoto, Japan; 4 Toranomon Hospital Kajigaya, Takatsu-ku, Kawasaki, Japan; 5 Department of Orthopedic Surgery, Saiseikai Niigata Kenoh Kikan Hospital, Kamisugoro, Sanjo, Niigata Japan; 6 Osteoporosis Center, Sapporo Maruyama Orthopaedic Hospital, Chuo-ku, Sapporo, Hokkaido Japan; 7 Department of Orthopaedic Surgery, Ken-Ai Memorial Hospital, Oaza Kimori, Onga-cho, Onga-gun, Fukuoka, Japan; 8 Bone and Joint Disease Center, Keiyu Orthopaedic Hospital, Akoudacho, Tatebayashi, Gunma Japan; 9 Okimoto Clinic, Kubi, Yutakamachi, Kure, Japan; 10 Soen Orthopaedics, Osteoporosis and Rheumatology Clinic, Okamoto, Higashinada-ku, Kobe, Hyogo Japan

**Keywords:** Osteoporosis, Once-daily vs. twice-weekly self-injection, Patient satisfaction, Teriparatide, Crossover comparison

## Abstract

**Introduction:**

Although bone anabolic agents such as teriparatide are effective for osteoporosis, satisfaction and adherence may vary by regimen. This multicenter study assessed long-term satisfaction, persistence, efficacy, and safety in postmenopausal women with primary osteoporosis treated with alternating daily and twice-weekly teriparatide over 52 weeks, followed by a final free-choice treatment period.

**Materials and methods:**

In a randomized, open-label, crossover study, 358 postmenopausal women at high risk for fracture were assigned to receive once-daily (20 µg) or twice-weekly (28.2 µg) subcutaneous teriparatide for 26 weeks, then crossed over to the alternative regimen for another 26 weeks. Afterwards, 233 patients entered a 52-week free-choice period under their preferred regimen.

**Results:**

Among the 233 patients entering the free-choice period, 162 chose twice-weekly and 71 chose daily teriparatide. Persistence at 104 weeks was 90.1% for twice-weekly and 88.7% for daily groups (p = 0.749). Overall and treatment satisfaction between groups did not differ significantly at 104 weeks (p > 0.05). Fracture incidence was low and similar (2.8% vs. 1.2%, p = 0.758). Patients in both groups showed significant increases in bone mineral density at L2–L4 and the femoral neck (p < 0.05). Adverse events were infrequent and non-severe.

**Conclusions:**

Patient satisfaction and efficacy were maintained with both teriparatide regimens over 104 weeks, and persistence improved during the patient-choice phase. Supporting patient preference may improve adherence to osteoporosis medications.

**Clinical trial registration:**

Japan Registry of Clinical Trials ID: jRCTs031210187.

## Introduction

Osteoporosis is a chronic skeletal disease characterized by low bone mineral density (BMD) and deteriorated bone microarchitecture, resulting in reduced bone strength and an increased risk of fractures [1]. Although currently available osteoporosis drugs efficiently improve BMD, the most important patient outcomes, such as the prevention of fractures, are inconsistent because of the heterogeneity in the magnitude of risk reduction for vertebral, hip, and clinical fractures between treatments in trials [2]. This heterogeneity may be due to the different baseline fracture risks of patients in these trials, the types or combinations of agents used, the types of treatment regimens, and patient adherence to treatment.

The availability of bone anabolic agents, combined with various treatment regimens, has led to a shift in the initial treatment of osteoporosis from antiresorptive to anabolic agents. Accumulating evidence suggests that patients at high risk of fracture should first be treated with bone anabolic agents [3, 4]. Moreover, it has been reported that BMD accrual is maximized when patients are initially given anabolic agents, followed by potent antiresorptive therapy [3]. In addition, a recent systematic review, network meta-analysis, and meta-regression analysis of randomized clinical trials in postmenopausal women demonstrated that bone anabolic agents are more effective than antiresorptive agents in reducing the incidence of vertebral and clinical fractures, regardless of mean age and baseline fracture risk [5].

Nonadherence and nonpersistence with treatment are particularly problematic in patients with osteoporosis because discontinuation of drug therapy rapidly increases the risk of fracture [6]. Treatment adherence can be improved by patient education, treatment preference, frequency, and ease of treatment [7]. Indeed, the Denosumab Adherence Preference Satisfaction study, a 24-month, randomized, crossover comparison study, demonstrated that postmenopausal osteoporosis patients were more adherent, compliant, and persistent with every 6-month subcutaneous injection of denosumab than with once-weekly tablets of alendronate and that treatment preference and satisfaction were higher with denosumab than with oral alendronate [8].

Teriparatide (TPTD) is a representative bone anabolic agent for the treatment of osteoporosis, and there are several different treatment formulations of TPTD in Japan. To evaluate patient satisfaction with two self-injection regimens, daily injection of TPTD (1/D-TPTD) and twice-weekly injection of TPTD (2/W-TPTD), we have conducted a randomized crossover (26 weeks for each period) comparison study [9]. The study consisted of 3 observational periods: an initiation period (0–26 weeks), a crossover period (27–52 weeks), and a free-choice period (53–104 weeks). We previously reported that neither overall patient satisfaction nor treatment satisfaction differed significantly between the two TPTD regimens during the initiation and crossover periods. However, patient satisfaction with the utility of the injection regimen had a trend in favor of 2/W-TPTD, suggesting that factors such as ease of preparation, handling, and use of the injection device, frequency of injections, and pain at the injection site may affect patient satisfaction and contribute to treatment adherence [9]. We also reported the results in the crossover period (27–52 weeks), showing a significantly higher preference for 2/W-TPTD (69.4%) than for 1/D-TPTD in the last crossover period [10].

The present report covers the final period of the study, during which patients were allowed to choose one of the treatment regimens based on their preference, and the treatment continued for an additional 52 weeks (free-choice period: 53–104 weeks). Here, we report patients’ satisfaction, persistence, efficacy, and safety.

## Materials and methods

### Study patients

This multicenter crossover study (Japan Registry of Clinical Trials ID: jRCTs031210187) was conducted at 39 centers in Japan between July 2021 and September 2023. Postmenopausal women with primary osteoporosis [11], aged 60 or older, at high fracture risk were eligible if they satisfied any of the following inclusion criteria: BMD < 60% of the young adult mean (YAM) or < − 3.3 standard deviations (SDs); ≥ 2 vertebral fractures (assessed by a semiquantitative method [12]) between the fourth thoracic vertebra (Th4) and fourth lumbar vertebra (L4); a grade 3 vertebral fracture; ≥ 1 vertebral fracture at Th4–L4 and BMD ≤ − 2.5 SD of YAM; or history of hip fracture. Patients with secondary osteoporosis, with bone loss due to diseases other than osteoporosis, on medications that may modify the TPTD effect, with experience of a self-injection device, or previous TPTD treatment were excluded. Details of the exclusion criteria have been described in our previous report [9].

### Study design

The protocol was approved by the Certified Review Board in the National Center of Global Health and Medicine. After providing their informed, written consent to participate, eligible patients were randomized into two groups. One group received a once-daily dose (20 µg self-injection) of teriparatide (1/D-TPTD group), and the other, a twice-weekly dose (28.2 µg self-injection) of teriparatide (2/W-TPTD group) for 26 weeks (initiation period). The dosing regimen was then switched, and treatment was continued for another 26 weeks (crossover period).

After completing the 52-week crossover study, patients were allowed to receive either a 1/D dose or 2/W dosing, depending on their preference, and the treatment continued for an additional 52 weeks (free-choice period). All patients received daily vitamin D supplementation (25 µg/day) throughout the study period. The study was conducted in accordance with the ethical principles outlined in the contemporary version of the Declaration of Helsinki, and was compliant with the Clinical Trial Act and related national ministerial orders, as well as all applicable regulations and ethical guidelines.

### Endpoints

Patient satisfaction was evaluated using a Patient Satisfaction Questionnaire [9]. The questionnaire comprises one question on overall satisfaction, two questions on the effectiveness of treatment, and 12 questions on the utility of the injection device with ease of use rated from “difficult” to “easy” on a 3-point or 6-point scale. The primary endpoint of the study was the degree of overall satisfaction at 26 weeks. The secondary endpoints were (1) overall patient satisfaction at 52 and 104 weeks, (2) patient satisfaction with treatment at 26, 52, and 104 weeks, (3) time course of patient satisfaction with the device utility at 26, 52, and 104 weeks, (4) preference of injection regimens when the patient was allowed to choose at 52 weeks, (5) persistence, and (6) efficacy of the treatment. Treatment efficacy was assessed by the number of incident fractures, changes in BMD, and quality of life (QOL) using the EuroQoL-5 Dimension (EQ-5D) scale, as well as pain, assessed by a visual analog scale (VAS). BMDs were measured at lumbar 1 to 4 (L1–L4) or 2 to 4 (L2–L4), femoral neck, and total hip by dual-energy X-ray absorptiometry. The safety of the treatment was evaluated based on the incidence, type, and severity of adverse events (AEs).

### Statistical analyses

Assuming a score difference of 0.7 between the two groups and an SD of 2.0, a sample size of 180 participants was required to achieve over 90% power with a two-sided alpha error of 5%. Therefore, accounting for an expected dropout rate of 10%, the target sample size was set at 200 subjects. Endpoints were analyzed in the full analysis set (FAS). All data are presented as the mean ± SD or frequency and percentage. The differences in baseline characteristics between the 1/D-TPTD and 2/W-TPTD groups were evaluated using a t test for continuous variables and a χ^2^ test used for categorical variables. The means and SDs of the patient satisfaction scores were determined, and differences between groups were assessed using a t test. The incidence of clinical fracture was evaluated using a Fisher test. A paired t test was used to assess time-dependent changes in BMD from baseline. Treatment dropout rate were summarized using Kaplan–Meier plots and evaluated with a log-rank test. Differences with p < 0.05 were considered significant. All statistical analyses were conducted using SAS software (version 9.4; SAS Institute, Cary, NC, USA).

## Results

**Fig. 1:**
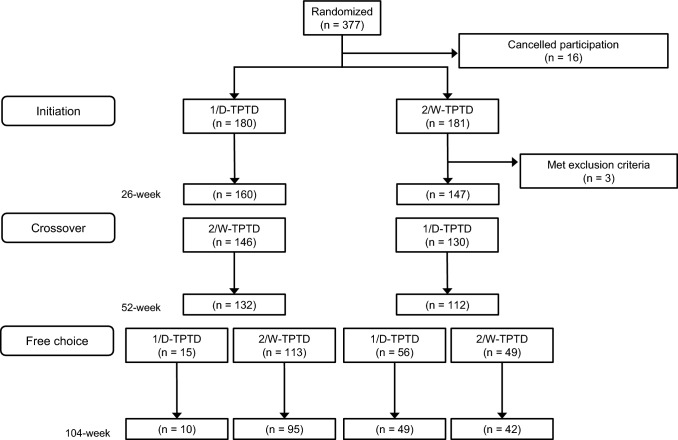
CONSORT flow diagram for the 3 observation periods. *TPTD* teriparatide, *1/D-TPTD* daily injection of TPTD, *2/W-TPTD* twice-weekly injection of TPTD

A CONSORT flow diagram for this study covering the 3 observation periods is presented in Fig. 1. We enrolled 358 patients (180 in the 1/D-TPTD group and 178 in the 2/W-TPTD group) in the initial 26-week period, after which the treatment regimen was switched and continued for a further 26 weeks (crossover period). After completion of the 52-week crossover period, a total of 233 patients were eligible to receive 1/D-TPTD or 2/W-TPTD regimens, depending on their preference. During the free-choice period, 71 patient participants chose 1/D-TPTD, and 162 chose 2/W-TPTD, receiving an additional 52 weeks of treatment until 104 weeks. For example, patients who received 1/D-TPTD for the first 26 weeks, 2/W-TPTD for the next 26 weeks, and then transitioned to 1/D-TPTD for the last 52-week free-choice period are abbreviated as 1-2-1 group patients.

### Patient characteristics

The baseline characteristics of patients who completed the 52-week crossover period and started free-choice period are shown in Table 1. No significant differences were found in characteristics of the 2 groups, confirming that the allocation was appropriate.

**Table 1:** Patient baseline characteristics after the crossover period

Item	1/D to 2/W-TPTD (n = 128)	2/W to 1/D-TPTD (n = 105)	p
Age, years	75.4 ± 7.1	75.0 ± 6.8	0.652
Age at menopause, years	49.9 ± 5.3	49.7 ± 3.5	0.718
Height, cm	150.5 ± 5.9	150.7 ± 6.3	0.874
Body weight, kg	50.2 ± 7.5	51.0 ± 8.9	0.504
BMI, kg/m^2^	22.2 ± 3.2	22.5 ± 3.8	0.574
BMD, T-score			
Lumbar spine (L2–L4)	− 2.44 ± 1.47	− 2.38 ± 1.41	0.784
Femoral neck	− 3.29 ± 0.80	− 3.21 ± 0.91	0.517
Total hip	− 2.55 ± 0.86	− 2.59 ± 1.12	0.815
Pain VAS score	29.0 ± 29.0	27.4 ± 30.9	0.675
EQ-5D utility score	0.89 ± 0.24	0.93 ± 0.20	0.242
Clinical fracture within 1 month			
Yes	34 (26.6%)	32 (30.5%)	0.510
No	94 (73.4%)	73 (69.5%)	
History of hip fracture			
Yes	14 (10.9%)	13 (12.4%)	0.732
No	114 (89.1%)	92 (87.6%)	
Complications			
Yes	73 (57.0%)	56 (53.3%)	0.572
No	55 (43.0%)	49 (46.7%)	
Previous treatment for osteoporosis			
Yes	48 (37.5%)	51 (48.6%)	0.089
No	80 (62.5%)	54 (51.4%)	

*EQ-5D* EuroQoL-5 Dimension, *TPTD* teriparatide, *1/D-TPTD* daily injection of TPTD, *2/W-TPTD* twice-weekly injection of TPTD, *VAS* visual analog scale

Nevertheless, several characteristics at the start of the free-choice period were significantly different between the 1/D-TPTD (n = 71) and 2/W-TPTD (n = 162) groups. The age was younger (73.8 ± 7.8 years vs. 75.8 ± 6.4, p = 0.046), the total hip BMD T-score was lower (− 2.85 ± 0.88 vs. − 2.46 ± 1.00, p = 0.046), and the VAS score for pain was lower (21.2 ± 29.3 vs. 31.4 ± 29.5, p = 0.016) in the 1/D-TPTD group than in the 2/W-TPTD group. No significant differences were found between the groups in other baseline characteristics.

### Persistence

During the 52-week free-choice period, the treatment persistence rates were 88.7% for the 1/D-TPTD group and 90.1% for the 2/W-TPTD group, respectively, with no significant difference observed between the 2 TPTD groups (p = 0.749).

**Fig. 2:**
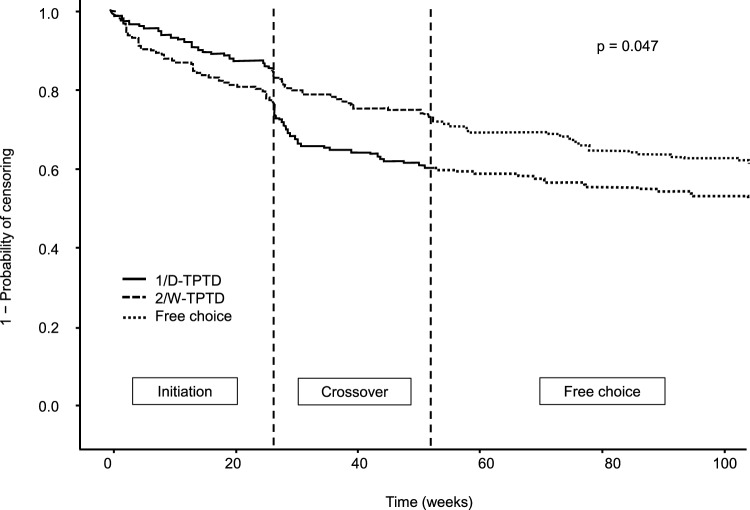
Time-dependent persistence during the 3 observation periods. *TPTD* teriparatide, *1/D-TPTD* daily injection of TPTD, *2/W-TPTD* twice-weekly injection of TPTD

Changes over time in the treatment dropout rate up to a total of 104 weeks are shown in Fig. 2. The significant difference between the 1/D-TPTD to 2/W-TPTD groups and the 2/W-TPTD to 1/D-TPTD groups observed at 52 weeks was maintained during the subsequent 52-week free-choice period and was still significant at 104 weeks (58.3% vs. 51.1%, p = 0.047).

### Endpoints

Table 2 summarizes the degree of overall patient satisfaction and satisfaction with treatment effectiveness at 104 weeks, revealing no significant difference between the 1/D and 2/W-TPTD groups. There were also no significant differences in these satisfaction levels between the groups at 26 and 52 weeks [9, 10].

**Table 2:** Patients’ overall satisfaction and patients’ satisfaction with treatment

Items	Question	104W (n = 233)		p
		1/D-TPTD (n = 71)	2/W-TPTD (n = 162)	
Overall satisfaction	How would you rate your satisfaction with this TPTD preparation? Please rate your satisfaction on a scale of 0 to 5 (0: dissatisfied to 5: satisfied)	4.3 ± 0.8	3.8 ± 1.1	0.202
		4.0 ± 1.1	3.6 ± 0.8	0.055
Satisfaction with treatment effectiveness				
Q1	Do you feel positive effects of the TPTD preparation being used? Please rate the effects on a scale of 0 to 5 (0: no to 5: yes)	3.6 ± 1.0	3.4 ± 1.5	0.654
		3.3 ± 1.4	3.0 ± 1.4	0.425
Q2	Do you feel improvement of lower or upper back pain with the TPTD preparation being used? Please rate the improvement on a scale of 0 to 5 (0: no to 5: yes)	3.0 ± 1.6	3.2 ± 1.6	0.709
		3.0 ± 1.4	2.6 ± 1.6	0.230

*TPTD* teriparatide, *1/D* once daily, *2/W* twice weekly

### Clinical fractures

The number and percentage of incident clinical fractures during the 52-week free-choice period were 2/71 (2.8%) in the 1/D-TPTD group and 2/162 (1.2%) in the 2/W-TPTD group, with no significant difference between the groups (p = 0.758).

### Effects on BMD

The percent changes from 52 to 104 weeks at L2–L4, the femoral neck, and total hip were 4.0 ± 5.1% (p < 0.001), 2.2 ± 5.0% (p = 0.018), and 1.5 ± 3.4% (p = 0.185) in the 1/D-TPTD group and 2.6 ± 4.7% (p < 0.001), 2.2 ± 7.5% (p = 0.011), and 2.1 ± 3.7% (p < 0.001) in the 2/W-TPTD group. No significant difference in percent changes of BMDs was observed between the 2 TPTD groups at 3 measurement sites.

**Fig. 3:**
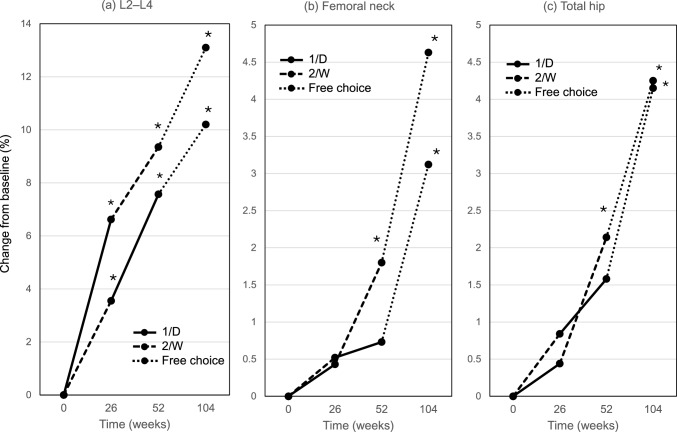
Changes of bone mineral densities. *Versus baseline. *1/D* daily injection of teriparatide, *2/W* twice-weekly injection of teriparatide

Figure 3a–c showed the changes in BMD from baseline at L2–L4, the femoral neck, and the total hip. L2–L4 BMD indicated a significant time-dependent increase from 26 to 104 weeks in both groups (p < 0.05). A significant increase in the femoral neck BMD and total hip was observed at 104 weeks in both groups.

### Adverse events

A summary of AEs observed during the 52-week free-choice period is shown in Table 3. AEs occurred in 1/71 (1.4%) patients in the 1/D-TPTD group and 4/162 (2.5%) patients in the 2/W-TPTD group (p > 0.99), but none of the AEs were severe.

**Table 3:** Adverse events reported during the free-choice period

	2/W-TPTD (n = 162)	1/D-TPTD (n = 71)
Nausea	2 (1.2%)	0
Feeling abnormal	1 (0.6%)	0
Decreased appetite	1 (0.6%)	0
Drug eruption	0	1 (1.4%)

*TPTD* teriparatide,*1/D-TPTD* daily injection of TPTD,* 2/W-TPTD* twice-weekly injection of TPTD

## Discussion

This study aimed to compare patient satisfaction with 2 TPTD regimens using a crossover method in Japan. In this study, osteoporosis patients at high risk for fracture were randomized to 1/D-TPTD or 2/W-TPTD treatment regimens and observed for 26 weeks (initiation period [9]). From 27 weeks, patients were crossed over to the treatment and observed for 52 weeks (crossover period [10]). From 53 weeks, patients were free to choose their treatment regimen by preference and were observed for an additional 52 weeks (free-choice period).

The important findings in this study are as follows:

1. The degree of overall patient satisfaction and the degree of satisfaction with the effectiveness of treatment at 26, 52 [9, 10], and 104 weeks were not different between TPTD groups. It was suggested that satisfaction may converge to a certain level at the end of the patient-approved treatment period. However, although differences in patients’ satisfaction regarding the usefulness of the injection device were observed at the beginning of the study, the differences disappeared by weeks 26 and 52 [9, 10]. These findings suggest that patients’ initial dissatisfaction with the injection device gradually fades as they become accustomed to it. Explaining the characteristics of treatment regimens and how to use the injection device before starting treatment may further increase satisfaction.
2. At the end of crossover period, significantly higher persistence was observed in the 1/D-TPTD to 2/W-TPTD group [10]. The treatment persistence rate during the last 52-week free-choice period was higher than that during the initial and crossover 52 weeks, and the rate was not significantly different between the 1/D-TPTD group and the 2/W-TPTD group during the free-choice period. This higher persistence may reflect the patients’ ability to select their preferred treatment regimen during that period.
  Persistence through 104 weeks was 50% or higher in both TPTD regimens. The persistence was relatively higher than previously reported values observed in clinical practice [13–16]. Conducting routine clinical tests or asking patients about their satisfaction with treatment may have a positive impact on their persistence.
3. In both TPTD groups, significant increases of 10% or more were observed in L2–L4 BMD at 26, 52 [9, 10], and 104 weeks. Femoral neck and total hip BMD increased significantly at 104 weeks in both TPTD groups. Osteoporosis requires long-term treatment, but treatment is often discontinued. The changes in BMD observed in this study suggest that continued treatment may lead to improved outcomes.
4. Incidence of clinical fracture and AEs were not significant differences between 1/D-TPTD and 2/W-TPTD at 26, 52 [9, 10], and free choice periods. The low frequency of AEs during the free-choice period may also have contributed to the high persistence rate. No harm in effectiveness and safety was found in switching TPTD treatments.

The present study has several limitations. First, significant differences in several baseline characteristics were observed between the two TPTD regimens among patients who completed the free-choice period. At the end of the crossover period, patients who selected 1/D-TPTD were younger than those who selected 2/W-TPTD, suggesting that younger patients may prefer the 1/D-TPTD regimen. However, there were no differences in patient characteristics at the end of the 52-week crossover observation period, confirming that appropriate treatment allocation was performed. Second, the fractures observed in this study were limited to clinical fractures, which were evaluated by attending physicians. Because the incidence of clinical fractures was lower than that of morphological vertebral fractures, information on fracture prevention may be an underestimate.

### Conclusions

Throughout this study, overall patient satisfaction and patients’ satisfaction with treatment were not different between the groups at 26, 52, and 104 weeks. Moreover, persistence was higher in the free-choice period. Furthermore, switching treatment regimens for TPTD does not affect efficacy or safety. This information will be helpful when selecting or switching TPTD treatments in clinical practice.

## Data Availability

The data that support the findings of this study are available from the corresponding author upon reasonable request.
